# Ovine macrophage identity and plasticity: novel insights into CSF-driven polarization and species-specific responses

**DOI:** 10.3389/fimmu.2025.1680086

**Published:** 2025-11-25

**Authors:** Yanina P. Hecker, Montserrat Coronado, Clara Hurtado-Morillas, David Arranz-Solís, Roberto Sánchez-Sánchez, Ángel Corbí, Luis Miguel Ortega-Mora

**Affiliations:** 1 SALUVET, Animal Health Department, Faculty of Veterinary Sciences, Complutense University of Madrid, Madrid, Spain; 2 INMIVET, Animal Health Department, Faculty of Veterinary Sciences, Complutense University of Madrid, Madrid, Spain; 3 Myeloid Cell Laboratory, Centro de Investigaciones Biológicas, CSIC, Madrid, Spain

**Keywords:** ovine macrophages, GM-CSF, M-CSF, immunophenotype, RNAseq

## Abstract

Macrophages (MØs) are pivotal immune cells exhibiting significant plasticity that has been widely studied in human and murine models. Granulocyte-macrophage colony-stimulating factor (GM-CSF) and macrophage colony-stimulating factor (M-CSF) are key regulators of macrophage differentiation from monocytes. In this study, we comprehensively investigated the immunophenotypic, functional, and transcriptomic profiles of ovine MØs differentiated with GM-CSF (GM-oMØs) or M-CSF (M-oMØs) to provide a more nuanced understanding of their activation states. After 7 days, GM-oMØs displayed a smaller, more varied morphology with lower cell yields compared to the larger, uniformly amoeboid M-oMØs. Immunophenotypically, M-oMØs showed significantly higher CD163 expression, consistent with human M-MØs, while CLEC5A was uninformative for differentiation. Transcriptomic analysis, complemented by qPCR and ELISA, revealed clearly distinct profiles, with GM-oMØs exhibiting a pronounced pro-inflammatory phenotype and showing significantly higher expression of 408 genes, mostly associated with interferon and inflammatory response pathways, a feature that aligns with the functional and phenotypic characteristics of human and mouse GM-MØ. Conversely, M-oMØs displayed a regulatory and anti-inflammatory profile, marked by a significantly higher expression of IL-10 and a set of 248 genes involved in cellular homeostasis. Notably, LPS stimulation dramatically shifted the M-oMØ phenotype toward a pro-inflammatory state, unequivocally demonstrating their substantial plasticity, and mirroring human M-CSF-polarized monocytes. Our findings fundamentally challenge the prevailing M1/M2 simplification in ovine macrophage biology and provide a robust foundation for selecting appropriate *in vitro* macrophage models for future investigations into ovine host defense and disease pathogenesis. This study demonstrated that M-oMØs exhibit greater plasticity, making them more suitable for pathogen-host interaction studies. Unlike GM macrophages, which already have a defined phenotype, M-oMØs more accurately reflect the dynamic immune response induced by a pathogen in the host.

## 1 Introduction

Macrophages (MØs) are central components of the mononuclear phagocyte system, playing critical roles in phagocytosis, antigen presentation, and immune response modulation (1). Their profound phenotypic and functional plasticity enables them to readily adapt to varied tissue microenvironments and adopt diverse activation states (2). This adaptability is shaped by factors such as their ontogeny (fetal origin *versus* monocyte-derived), specific tissue location, and microenvironmental influences (1, 2).

The sequential and finely tuned pro- and anti-inflammatory functions of MØs are essential for effective tissue repair and the return to homeostasis during inflammation. Granulocyte-macrophage colony-stimulating factor (GM-CSF) and macrophage colony-stimulating factor (M-CSF) are primary regulators of monocyte differentiation into MØs (3, 4). Research in human and murine models consistently shows that GM-CSF-exposed MØs (GM-MØs) develop increased pro-inflammatory activity, adopting the characteristic phenotype and gene expression pattern of lung alveolar MØs and anti-tumoral macrophages (2, 3, 5–7). Conversely, M-CSF drives MØs (M-MØs) toward an anti-inflammatory phenotype (2, 5, 8). Notably, human studies reveal a more nuanced picture, where TLR7-activated M-CSF MØs can demonstrate augmented pro-inflammatory responses and stronger neutrophil recruitment (9). Hamilton (10) proposed that a constant level of M-CSF is crucial for maintaining M-MØs in a homeostatic, resting condition, whereas a local and temporal increase in GM-CSF during inflammation shifts GM-MØs into an inflammatory state. Moreover, Rodriguez et al. (4) highlighted that M-MØs are less differentiated than GM-MØs, presenting an intermediate phenotype between monocytes and GM-MØs. This work also found that GM-CSF stimulation of monocytes induces a pro-inflammatory phenotype with reduced plasticity, indicating a terminal differentiation state (4).

In veterinary medicine, the study of MØ polarization has gained attention due to its relevance in developing alternative *in vitro* models for host-pathogen interactions (11–14). The importance of *in vitro* models using target cells lies in their ability to study host-specific immunopathology, adhering to the 3Rs principle by replacing animal experimentation. In mice, similarly to humans, bone marrow-derived MØs grown in M-CSF or GM-CSF have been reported to exhibit properties akin to M1 and M2 cells, respectively (5). More recently, Li et al. (13) conducted a transcriptomic study using porcine bone marrow-derived MØs stimulated with M-CSF or GM-CSF, demonstrating that porcine M1 and M2 MØs share consistent gene signatures with human and mouse MØs phenotypes. Nevertheless, despite these advancements, there are a limited number of studies in farm animals utilizing *in vitro* monocyte-derived MØ models, with most investigations circumscribing macrophage polarization exclusively to a GM-CSF-induced M1-like (pro-inflammatory) phenotype (11, 12, 15).

For instance, García-Sanchez et al. (11) employed GM-CSF-differentiated bovine MØs to investigate the ability of *Neospora caninum* isolates to infect bovine MØs. Furthermore, Queval et al. (14) utilized bovine GM-CSF-differentiated MØs to investigate the pathogen and host factors driving multinucleated cell formation in response to human and animal-adapted tubercle bacilli. Likewise, in ovine studies, Arteche-Villasol et al. (16) compared two technical protocols for generating GM-CSF-differentiated ovine MØs (oMØs). This approach was subsequently applied by Vallejos et al. (12) to examine how the genetic variability of *Toxoplasma gondii* strains affected different phenotypic traits. Importantly, M-MØs have been rarely employed for *in vitro* veterinary studies (13, 17–19). Given the extensive metabolic and functional plasticity observed in human MØs, further investigation into M-MØs compared to GM-MØs in other species is warranted, especially considering their potential as alternative experimental models. To gain insights into the biology of these innate immune cells in sheep, we comprehensively analyzed the phenotypic and functional heterogeneity of M-oMØs and GM-oMØs, including a high-resolution transcriptomic study to determine the molecular bases governing the differences between these two cell types. We provide evidence that M-oMØs exhibit an anti-inflammatory and regulatory phenotype, whereas GM-oMØs display a defined pro-inflammatory phenotype. Besides, we demonstrated that M-oMØs have a substantial plasticity and resemble human M-CSF-dependent monocyte-derived macrophages. Therefore, an *in vitro* model of M-oMØs would be more appropriate for virulence or vaccine studies that focus on understanding the specific immune response a pathogen activates. Conversely, using GM-oMØs could lead to erroneous conclusions because these cells are already activated and have a defined phenotype, which may misrepresent the initial host-pathogen interaction.

## 2 Materials and methods

### 2.1 Separation of mononuclear cells and phenotypic analysis of monocytes

A 500 mL whole blood sample was collected from 3 healthy adult non-pregnant sheep of Rasa Aragonesa breed, via venipuncture of the external jugular vein using a blood-bag system with citrate phosphate dextrose adenine-1 (CPDA-1) (Teruflex^®^; Terumo, Tokio, Japan). Peripheral blood mononuclear cells (PBMCs) were isolated from the buffy-coat fraction by density gradient centrifugation using Histopaque 1077 (Sigma-Aldrich, USA), as previously described (16). The interphase containing ovine monocytes was washed and resuspended in cold PBS/EDTA (2 mM EDTA, pH 8) supplemented with 0.05% bovine serum albumin (BSA) (Thermo Scientific, Belgium) for subsequent flow cytometry analysis.

Monocyte surface marker expression was determined using single and multiple labelling panels from three individual animals. Monoclonal antibodies against the following molecules were utilized: CD14, CD16, CD172a, MHC Class II, CD163, and CD11b. A comprehensive list of all antibodies used for subset analysis is provided in **Supplementary Table 1**.

For flow cytometry, PBMCs were initially resuspended at a density of 2 × 10^6^ cells/mL in cold PBS supplemented with 2% fetal calf serum (FCS) (Pan Biotech, Germany) (referred to as PBS-2% FCS). A volume of 100 µL per well of this cell suspension per well was transferred to a V-bottom 96-well plate and pelleted by centrifugation at 1,300 rpm for 3 minutes at 4°C. After aspirating the supernatant, cells were incubated with 50 µL of diluted antibody (1:100) or 50 µL of PBS-2% FCS (for controls) for 30 minutes on ice, protected from light. Following incubation, samples were washed with PBS-2% FCS before adding BD Cytofix™ fixative (BD Bioscience, USA). The percentage of positive cells and mean fluorescence intensity (MFI) for each marker were measured using a Becton Dickinson FACSCalibur cytometer (BD Bioscience, USA). Data analysis was performed using FlowJo V10 software (FlowJo, LLC, USA).

### 2.2 Generation of ovine monocyte-derived macrophages *in vitro*

Following PBMC isolation, monocytes were purified via positive magnetic selection using mouse anti-human CD14-coated microbeads (Miltenyi Biotec Ltd., USA), strictly adhering to the manufacturer’s guidelines (12, 16). The identity and purity (≥95%) of the isolated monocytes were subsequently confirmed by flow cytometry using a mouse anti-human CD14 antibody (**Supplementary Table 1**).

Purified CD14+ monocytes were then seeded into 12-well culture plates at a density of 0.5 × 10^6^ cells/mL in 2 mL of RPMI-1640 medium (Cytiva, Hyclone, USA). This medium was supplemented with 10% fetal calf serum (FCS), 100 IU/mL penicillin, 100 µg/mL streptomycin, and 50 µM β-mercaptoethanol (Merck Millipore, USA), collectively referred to as complete medium (CM). Monocyte cultures were maintained for 7 days at 37°C in a 5% CO_2_ atmosphere. During this period, CM was supplemented with either 10 ng/mL recombinant ovine M-CSF (Kingfisher Biotech, USA) to generate M-oMØs, or 100 ng/mL recombinant ovine GM-CSF (Kingfisher Biotech, USA) to generate GM-oMØs. Cytokines were replenished every 2 days at the same concentration. For oMØ activation, cells were treated with 10 ng/mL *Escherichia coli* O111:B4 strain-TLR4 ligand (LPS-EB Ultrapure, InvivoGen, USA) (9). All assays were performed in triplicate for each condition, using cells from three different animals across independent experiments.

### 2.3 Morphological description of M and G-oMØs

After 7 days in culture, the morphological features of M-oMØs and GM-oMØs were characterized using an inverted optical microscope (Nikon Eclipse TE 100). Observations focused on changes in cellular shape and granularity. Cell yield on day 7 was quantified for both M-oMØs and GM-oMØs by manual counting with a Neubauer chamber after Trypan blue staining. To measure cellular area, an inverted microscope (Nikon Eclipse TE 200) coupled with NIS Elements V.5.30.04 imaging software (Nikon) was employed.

### 2.4 Immunophenotypic and functional characterization of M and G-oMØs

#### 2.4.1 Flow cytometry for surface marker expression

Surface expression of CD14, CD16, MHC Class II, CD80, CD86, CD172a, CD11b, CD163, and CLEC5A on M-oMØs and GM-oMØs was determined by flow cytometry after 7 days of culture. Antibodies used are detailed in **Supplementary Table 1**. The methodology followed previously described protocols (see section 2.1) with minor modifications. Prior to antibody incubation, non-specific antibody binding was blocked by adding TruStain FcX™ antibody (Clone 96, BioLegend, San Diego, CA, USA). After immunostaining, samples were washed with PBS, and the viability dye 7-AAD (Biolegend, CA, USA) was added 5 minutes before flow cytometry analysis. For each sample, a minimum of 2 × 10^5^ viable events per sample were acquired on a Becton Dickinson FACSCalibur cytometer and analyzed using FlowJo software.

#### 2.4.2 RNA extraction and cDNA synthesis for gene expression analysis by qPCR

To complement the immunophenotypic characterization, mRNA expression levels of cytokines and chemokines were determined by quantitative real-time PCR (qPCR) in unstimulated M-oMØs and GM-oMØs after 7 days of culture. For this, oMØs were recovered by scraping and centrifugation at 300 × g for 10 minutes at 4°C. The resulting cell pellets were stored at -80°C until RNA extraction. Total RNA was extracted using the RNeasy Mini Kit (Qiagen, Germantown, MD) according to the manufacturer’s recommendations. RNA integrity was assessed by 1% agarose gel electrophoresis, and RNA concentrations were determined using an Epoch microvolume spectrophotometer system (BioTek^®^ Instruments, USA). RNA samples were reverse transcribed into cDNA using the SuperScript VILO cDNA Synthesis Kit (Invitrogen, UK), following the manufacturer’s protocol.

PCR was performed using the 7500 Fast Real-Time PCR System (Applied Biosystems, USA) with the Power SYBR^®^ Green PCR Master Mix (Applied Biosystems, USA), according to the manufacturer’s instructions. PCR amplification reactions contained 12.5 µL Power SYBR^®^ Green PCR Master Mix, 10 pmol of each primer, and 5 µL of diluted cDNA in a final volume of 25 μL. Primer sequences for the amplification of *IL-10*, *TNF-α*, *IL-6*, *IL-23*, *IL-12p40*, *IL-1β*, *TGF-β*, *CCL22*, *CCL24*, *CXCL10*, *GAPDH*, and *β-actin* genes have been previously published and are listed in **Supplementary Table 2**. Primer sequences for CCL17 were designed using Primer3Plus software (20), and their chromosomal and mRNA sequences were verified using BioEdit Sequence Alignment Editor v.7.1.3 (Tom Hall, Ibis Therapeutics, Carlsbad, CA, USA). β-actin and GAPDH served as housekeeping genes, yielding comparable Ct values across all samples. The linearity and efficiency of qPCR amplification for each primer pair were determined using a standard curve generated from a serial dilution of cDNA pooled from all samples. Relative quantification of gene expression was performed using the comparative threshold cycle (2^-ΔCt^) method (21). Product sizes were confirmed by agarose gel electrophoresis, and specificity of amplification was verified by melting curve analysis.

### 2.5 Transcriptomic analysis

Total RNA was extracted from untreated M-oMØs and GM-oMØs, as well as from LPS-treated M-oMØs (4 hours post-stimulation). All samples were obtained from three independent donors after 7 days of culture using the RNeasy Mini Kit (Qiagen, Germantown, MD). RNA purity and concentration were determined at 260/280 nm using an Agilent 2100 Bioanalyzer (Agilent, Santa Clara, CA, USA) with an Agilent RNA 6000 Nano Reagents Part 1 test kit (Agilent, Santa Clara, CA, USA). Samples were then subjected to sequencing on a BGISEQ-500 platform (https://www.bgitechsolutions.com). RNA-seq data were deposited in the European Nucleotide Archive (ENA) under project accession PRJEB93905.

Raw reads underwent filtering to remove low quality sequences, adapter contamination, and unknown bases, yielding clean reads. These clean data were aligned to the *Ovis aries* genome (Reference Genome Version: GCF_000298735.2_Oar_v4.0) using Bowtie2 (v2.3.4.3) (22). Gene expression quantification was performed using RSEM (v1.3.1) software (23).

Differential gene expression analysis was conducted using the DEGseq package (24). Differentially expressed genes (DEGs) were identified based on a threshold of Q-value ≤ 0.05 and ∣Log2 FC∣≥1. DEGs were further analyzed by overrepresentation analysis of annotated gene sets using ENRICHR (http://amp.pharm.mssm.edu/Enrichr/) (25). Enriched terms were considered significant if their Benjamini-Hochberg-adjusted p-value was < 0.05.

Raw counts were normalized to Transcripts Per Million (TPM). The TPM-normalized expression matrix was subsequently used for gene set enrichment analysis (GSEA) using the GSEA software (http://software.broadinstitute.org/gsea/index.jsp) (26) and the Hallmark gene set from the Molecular Signatures Database (MSigDB v2024.1) (27). Pathways with a false discovery rate (FDR) < 0.25 were considered significant.

### 2.6 Cytokine detection in culture supernatants

To further characterize the functional profiles of M-oMØs and GM-oMØs, cells were treated with 10 ng/mL LPS or left unstimulated for 16 hours on the seventh day of culture. Subsequently, the supernatants (SNs) from both treated and untreated GM-oMØs and M-oMØs were collected and analyzed. The production of soluble factors in these SNs was quantified using enzyme-linked immunosorbent assay (ELISA) kits for TNF-α (Cloud-Clone Corp^®^, Texas, USA) and IL-10 (MyBioSource^®^, CA, USA), according to the manufacturers’ recommendations.

### 2.7 Statistical analysis

Statistical analyses for the morphological and immunophenotypic studies were performed using GraphPad Prism v.8.0.1 software (San Diego, CA, USA). Comparisons of means were conducted using either an unpaired Student’s t-test or Kruskal-Wallis test, followed by Dunn’s multiple-comparison test where appropriate. In all analyses, a p-value < 0.05 was considered statistically significant.

## 3 Results

### 3.1 Sub-populations of ovine blood monocytes

**Figure 1:**
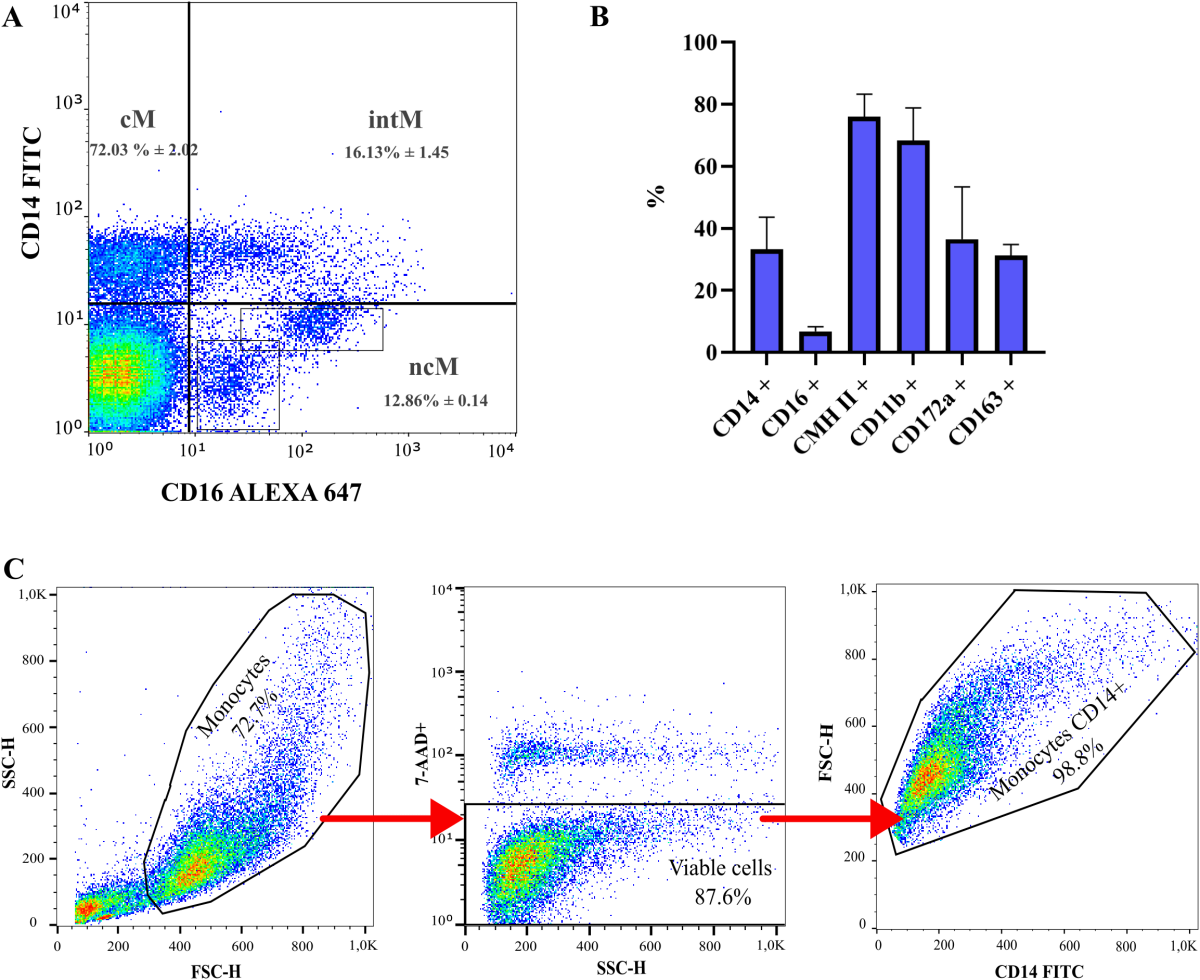
Sub-populations of ovine blood monocytes determined by flow cytometry. **(A)** Gating strategy of ovine monocyte subsets based on relative CD14 and CD16 expression. Dot plots of CD14 and CD16 expression display classical monocytes (cM) [% of MNC: 38.6 ± 7.30; Cells/mL blood (x 10^5^): 30.30 ± 5.73], intermediate monocytes (intM) [% of MNC: 8.51 ± 2.42; Cells/mL blood (x 10^5^): 6.68 ± 1.90] and nonclassical monocytes (ncM) [% of MNC: 6.71 ± 1.33; Cells/mL blood (x 10^5^): 5.27 ± 1.04]. N = 3. MNC: Mononuclear cells population. **(B)** Surface markers of ovine monocytes by flow cytometry. N = 3. %: Frequency of positive cells. **(C)** Gating strategy of purified ovine monocyte subsets based on relative CD14 expression after purification with immunomagnetic columns.

Following the isolation of peripheral blood mononuclear cells (PBMCs) and prior to the purification of CD14+ monocytes, ovine monocytes were immunophenotypically characterized. Ovine monocytes were identified based on their expression of CD14 and CD16. Comparative analysis of the expression of CD14 and CD16 allowed the identification of three monocyte subsets: classical CD14+/CD16- (72% ± 2.0%), intermediate CD14+/CD16+ (13% ± 5.0%) and nonclassical CD14-/CD16+ (13% ± 0.6%) (**Figure 1A**). Additionally, CD16-positive sub-populations exhibited varying fluorescence intensities and complexity, while CD14 expression appeared more uniform (**Figure 1A**). The percentages of CD172a, MHC Class II, CD163, and CD11b expression were also evaluated (**Figure 1B**). After purification using immunomagnetic columns, the purity of CD14+ cells consistently exceeded 95% (**Figure 1C**). This characterization confirmed that, like their human and mouse counterparts, ovine blood monocytes display a distinct three-subset distribution according to CD14 and CD16 expression, where CD16 expression is more heterogeneous than CD14. Based on this finding, we proceeded to isolate a highly enriched CD14+ monocyte subset for subsequent macrophage differentiation experiments, similar to what has previously done on monocytes from ruminants (16).

### 3.2 Phenotypic and functional features of GM- and M-oMØs

#### 3.2.1 Morphological description of GM- and M- oMØs

**Figure 2:**
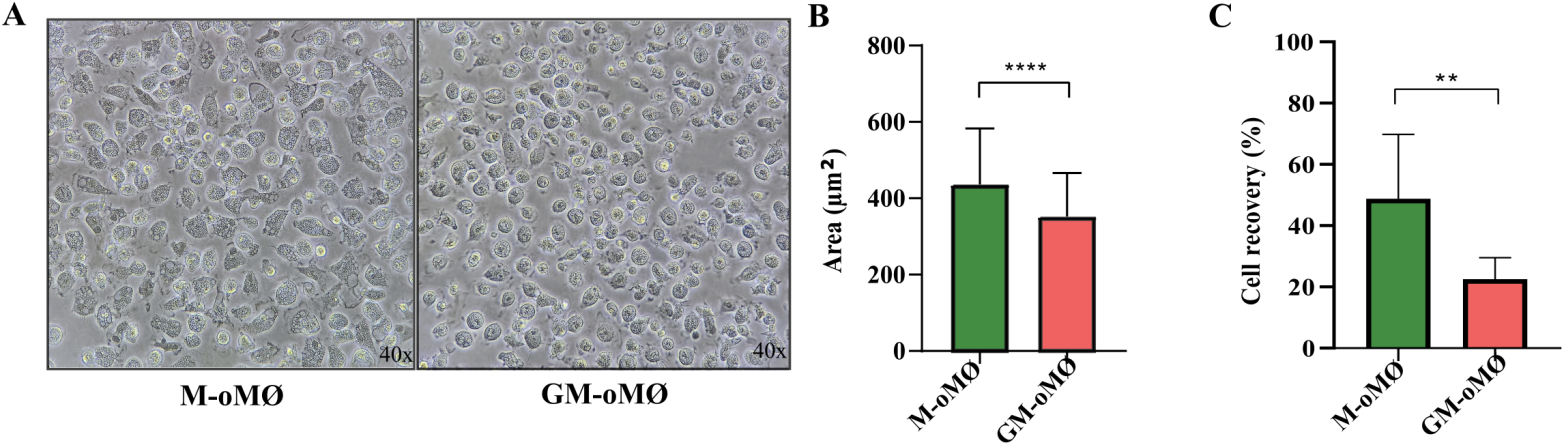
Characteristics of CD14+ monocyte-derived oMØs. **(A)** Morphology of CD14+ monocyte-derived oMØ after 7 days of culture with M-CSF (left image) or GM-CSF (right image). Original magnification ×40. **(B)** Area of CD14+ monocyte-derived oMØ cultured for 7 days with GM-CSF or M-CSF, expressed as the mean ± SD of three independent donors. **(C)** Cell recovery of CD14+ monocyte-derived oMØ cultured for 7 days with GM-CSF or M-CSF, expressed as the mean percentage (relative to initial seeding) ± SD of three independent donors. **p<0.01, ****p<0.0001.

To assess the ability of ovine monocyte to differentiate along the macrophage (oMØ) lineage, they were cultured in the continuous presence of either M-CSF or GM-CSF. After 7 days of culture, GM-oMØs displayed a more varied morphology, including both rounded and fusiform cells, and were significantly smaller than M-oMØs (**Figures 2A, B**). Conversely, M-oMØs exhibited an amoeboid-like morphology with granular cytoplasm and a uniform size (**Figure 2A**). Furthermore, the cell yield at the end of the 7-day culture period was higher for M-oMØs compared to GM-oMØs (**Figure 2C**). Overall, while M-oMØs showed a more uniform amoeboid morphology and higher yield, GM-oMØs were characterized by a more heterogeneous, often fusiform appearance and smaller size.

#### 3.2.2 Immunophenotype of GM- and M- oMØs

##### 3.2.2.1 Expression of markers by flow cytometry in M-oMØs and GM-oMØs

**Figure 3:**
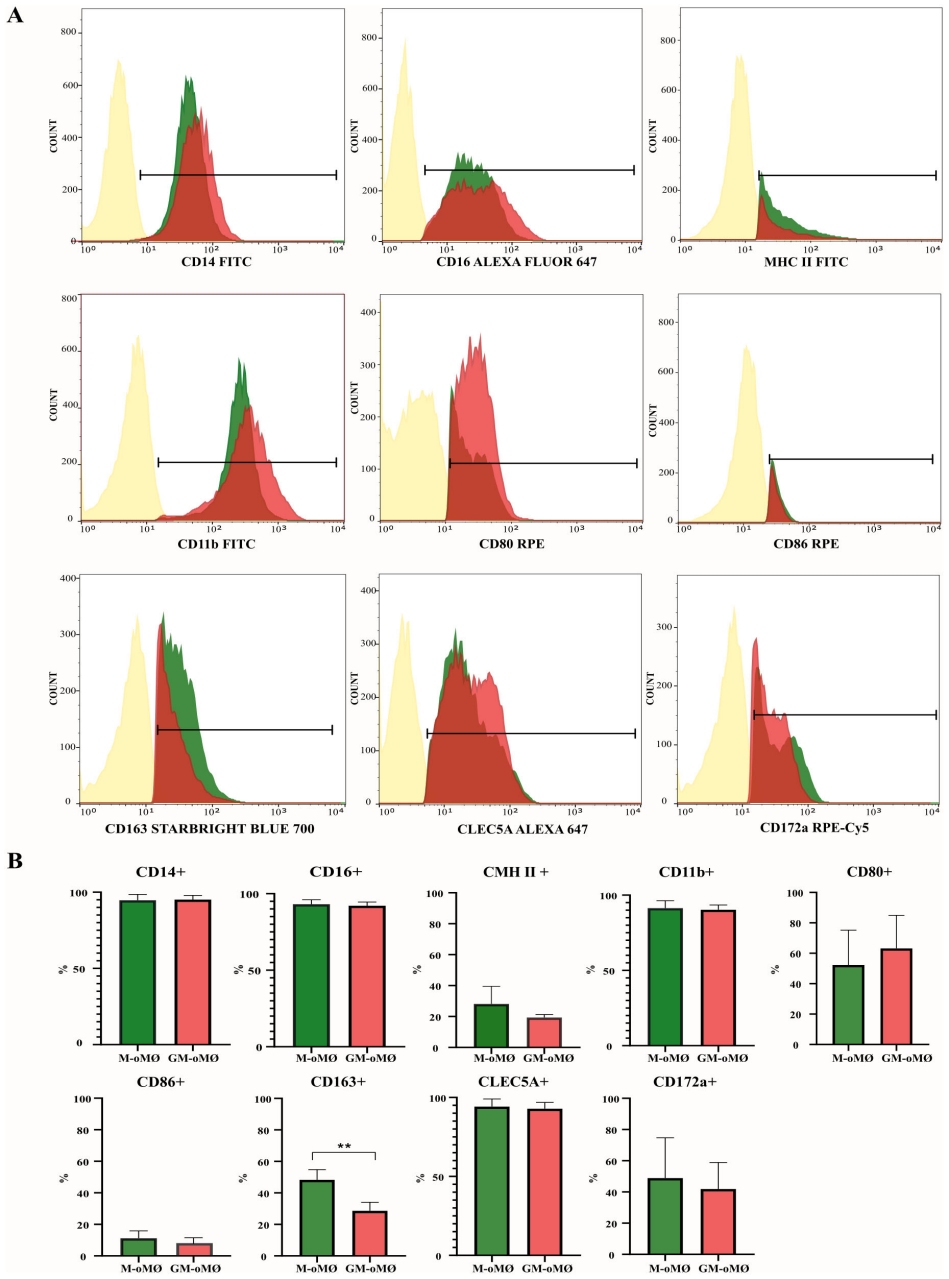
Flow cytometric analysis of M- and GM-oMØs. **(A)** Histograms represent the percentage of positive cells and mean fluorescence intensity (MFI) of CD14, CD16, MHC class II, CD11b, CD80, CD86, CD172a, CD163 and CLEC5A markers, as determined by flow cytometry in non-stimulated oMØ after 7 days of culture with GM-CSF (red) or M-CSF (green). Autofluorescence of unstained oMØs is represented in yellow. Histograms are representative of three independent experiments. **(B)** Difference in expression markers in M- and GM-oMØs. Data are presented as mean percentage (%) ± SD. **p<0.01.

**Figure 3** presents the expression levels of evaluated markers in M-oMØs and GM-oMØs. High expression of CD14, CD16, and CD11b markers was observed in both GM-oMØs and M-oMØs, although the mean fluorescence intensity (MFI) for CD14+ cells was higher in GM-oMØs (individual replicate data are available in **Supplementary Table 3**). Consistent with human studies, the CD163 marker exhibited significantly higher expression in M-oMØs (p < 0.05) (**Figure 3B**). Interestingly, and in contrast to human findings, CLEC5A expression was similarly high in both M-oMØs and GM-oMØs, suggesting it is not an informative marker for distinguishing these oMØs subtypes in the present study. Furthermore, the expression of MHC Class II, CD80, and CD86 markers was low in both cell types, likely owing to their basal (non-activated) state. Collectively, these immunophenotypic findings reveal that while CD163 is a distinguishing marker for M-oMØs in sheep, mirroring human observations, and that the high expression of CLEC5A in both macrophage types represents a notable divergence from human macrophage immunophenotypes, highlighting a species-specific difference.

##### 3.2.2.2 Cytokine and chemokine expression in M-oMØs and GM-oMØs

**Figure 4:**
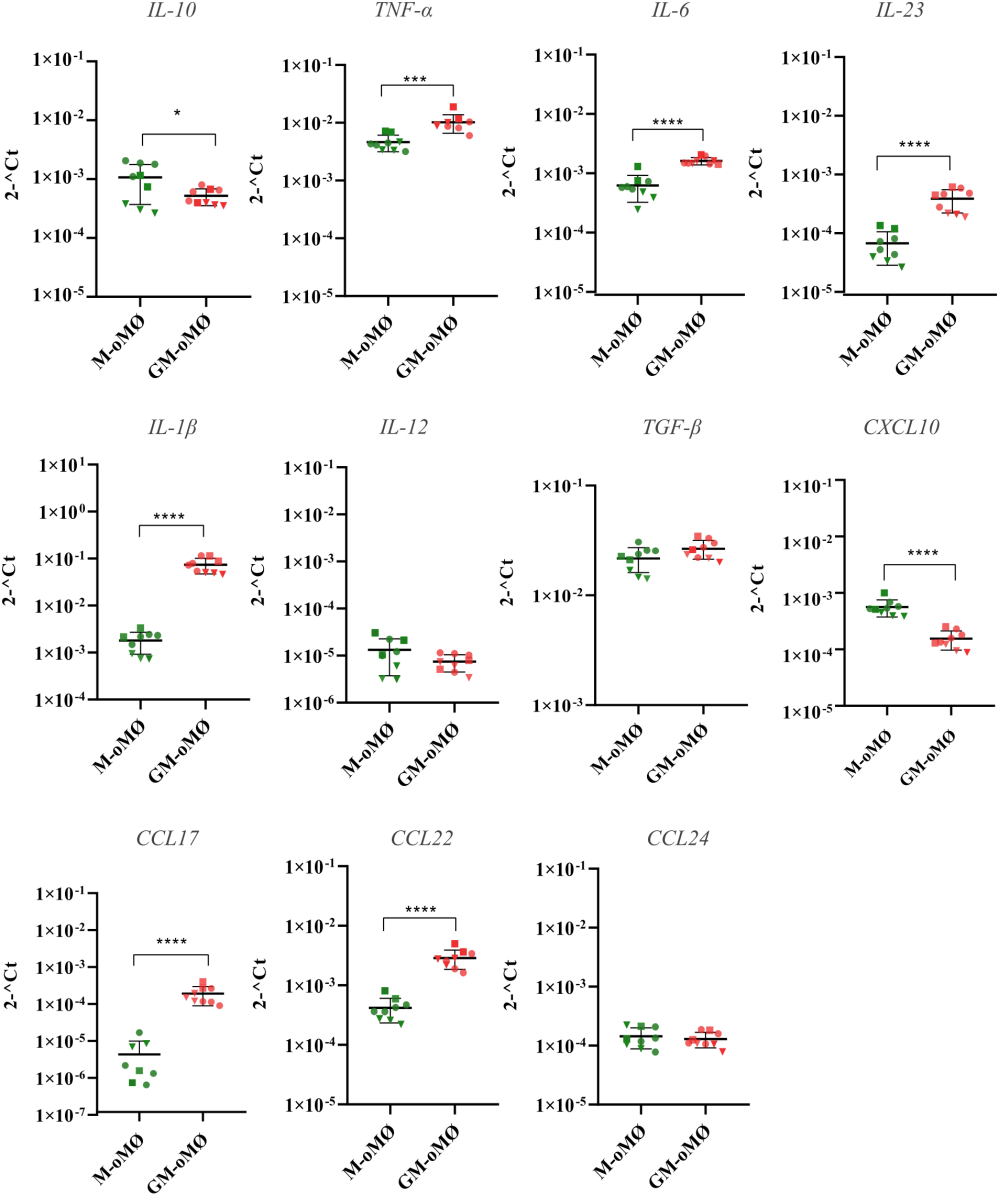
Cytokine and chemokines expression levels evaluated by qPCR in unstimulated M and GM-oMØs. Results are based on three experimental replicates with three different animals (each replicate from each animal is represented by a symbol). Scatter-plot graphs represent relative cytokine expression levels in both oMØs types. Horizontal lines represent median values for each group. *p<0.05, ***p<0.001, ****p<0.0001.

**Figure 4** presents the levels of RNA for various cytokines and chemokines in M-oMØs and GM-oMØs, as determined by q-PCR. In the basal state, the mRNA levels of the pro-inflammatory cytokines *TNF-α*, *IL-23*, *IL-6*, and *IL-1β* were significantly higher in GM-oMØs (p < 0.05). Similarly, the levels of chemokines *CCL22* and *CCL17* were elevated in GM-oMØs relative to M-oMØs (p < 0.05). However, no significant differences were observed in the mRNA levels of *IL-12A* between both subtypes (p > 0.05). Conversely, RNA levels for *IL-10* and *CXCL10* were higher in M-oMØs (p < 0.05), whereas *TGF-β* and *CCL24* RNA levels were similar in both cell types (p > 0.05). Overall, these findings reveal a clear pro-inflammatory profile in basal GM-oMØs, characterized by higher expression of genes coding for key pro-inflammatory cytokines and chemokines, while M-oMØs show a preferential expression of the gene encoding the paradigmatic anti-inflammatory and regulatory *IL-10* cytokine, thus supporting clearly distinct functional orientations for M-oMØs and GM-oMØs subsets.

### 3.3 Transcriptional differences between GM- and M-oMØs

We next proceeded to elucidate the molecular basis underpinning the phenotypic and functional differences between GM-oMØs and M-oMØs. To this end, we compared the transcriptional signatures of both GM-oMØ and M-oMØ. A total of six samples were sequenced using the DNBSEQ platform, yielding an average of 13.47 GB of data per sample. An average of 90 million reads was obtained for each sample, and a total of 18,781 genes were detected after alignment with the reference genome (GCF_000298735.2_Oar_v4.0). The integrity of the starting biological material and the absence of significant deviations in the sequencing processes were verified by data quality controls.

**Figure 5:**
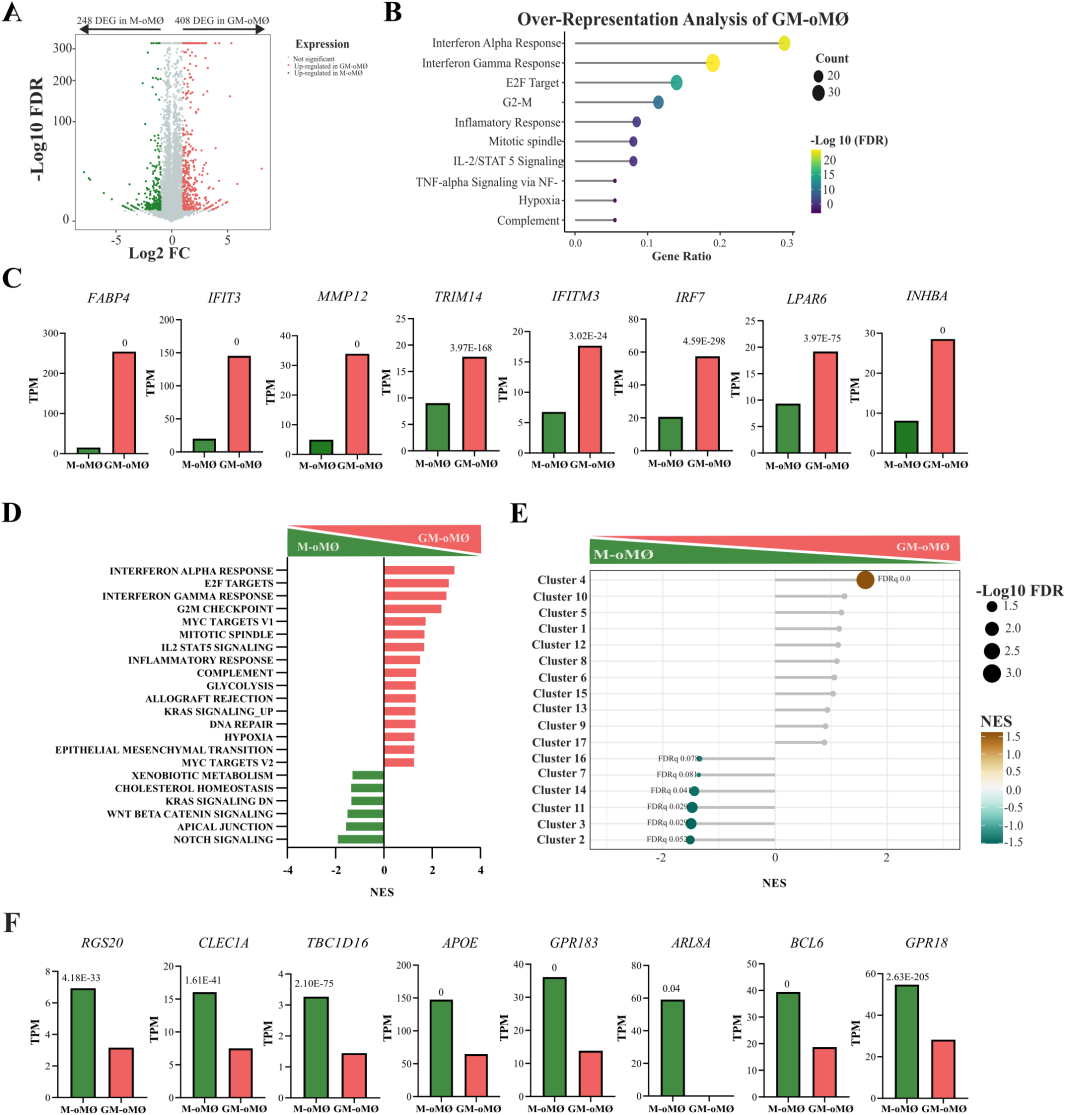
Transcriptional analysis of M- and GM-oMØs after 7 days of culture. **(A)** Volcano plot representing differentially expressed genes (DEGs) in GM- oMØs (red) and M-oMØs (green) (|Log2 FC| ≥ 1, Q value ≤ 0.05) with a square transformation applied to the y-axis. **(B)** Enrichment analysis of DEGs in GM-oMØs. The top 10 most significant terms are shown. Dot size indicates the number of DEGs in each term, and the color gradient represents the -Log 10 (FDR). **(C)** Relative mRNA levels (TPM) of selected DEGs in GM-oMØs as determined by RNA-Seq on three independent samples. Q-value is shown in each case. **(D)** Gene Set Enrichment Analysis (GSEA) of Hallmark gene sets based on the transcriptomes of GM-oMØs and M-oMØs. Normalized Enrichment Scores (NES) are indicated for each gene set. **(E)** GSEA of human MoMac-VERSE gene sets based on the ranked comparison of GM-oMØs *vs* M-oMØs transcriptomes. FDR values are indicated for significantly enriched gene sets. Non-significant gene sets are shown as grey dots. The color gradient indicates NES. **(F)** Relative mRNA levels (TPM) of selected DEGs in M-oMØs as determined by RNA-Seq on three independent samples. Q-value of the comparison is shown in each case.

Among the total ovine genes identified, 657 differentially expressed genes (DEGs) (Q-value ≤ 0.05 and ∣Log2 FC∣≥1) were identified when comparing the transcriptomes of GM-oMØs and M-oMØs (**Supplementary Table 4**). Of these, 408 genes were upregulated in GM-oMØs (**Figure 5A**). Enrichment analysis using the Hallmark gene sets revealed that these DEGs were significantly enriched in pathways related to Interferon gamma response, Interferon alpha response, E2F Targets, G2-M Checkpoint, inflammatory response, mitotic spindle, IL-2/STAT5 signaling, Tumor Necrosis Factor alpha signaling via NF-Ƙβ, hypoxia, complement, and angiogenesis (adjusted p-value < 0.05) (**Figure 5B**). Key genes associated with inflammatory response included *FABP4*, *IFIT3*, *MMP12*, *TRIM14*, *IFITM3*, *IRF7*, *LPAR6*, and *INHBA* (**Figure 5C**). Similarly, GSEA analysis indicated that pathways associated with Interferon alpha response, E2F target, Interferon gamma response, and G2M checkpoint were the most significantly enriched in the GM-oMØs gene profile (FDR < 0.05) (**Figure 5D**). Additional GSEA utilizing the human MoMac-VERSE gene sets, as defined by Mulder et al. (2), further supported the inflammatory profile of GM-oMØs. Specifically, GM-oMØs showed an increased expression of the gene set defining the MoMac-VERSE macrophage subset identified as Cluster #4 (ISG_Mo) (FDR < 0.05) (**Figure 5E**) associated with proinflammatory M1 genes in humans (2). Overall, the transcriptomic analysis of GM-oMØs reveals a strong enrichment of pro-inflammatory and cell proliferation pathways.

Conversely, 248 genes were found to be upregulated in M-oMØs (**Figure 5A**), although their gene ontology analysis did not reveal any significant enrichment. Nevertheless, many of these DEGs have been previously associated with the maintenance and regulation of cellular homeostasis, such as *RGS20*, *CLEC1A*, *TBC1D16*, *APOE*, *RALGPS2*, *GPR183*, *ARL8A*, *BCL6*, and *GPR18* (**Figure 5F**). Furthermore, GSEA analysis revealed significant enrichment in pathways associated with Notch signaling, Apical Junctions, and WNT Beta Catenin signaling (FDR < 0.05) (**Figure 5D**). Finally, comparative analysis with the MoMAC-VERSE (2) showed a significant enrichment of gene sets defining MoMac-VERSE macrophage subsets identified as Cluster#2 (HES_1), #3 (TREM2), #11 (Mac), #14 (DC2/DC3), #7 (Mac), and #16 (C1Q Mac) (FDR < 0.1) (**Figure 5E**). Interestingly, Clusters #2 (HES_1) has been identified as long-term resident macrophages and Cluster #16 and #17 as tumor-associated macrophages with an M2-like signature (2). Therefore, the transcriptomic analysis of both oMØs subtypes further supports GM-oMØs to exhibit a robust pro-inflammatory profile, while M-oMØs preferentially display a regulatory or anti-inflammatory transcriptional signature.

### 3.4 GM- and M oMØs secrete soluble factors that dictate their phenotypes

**Figure 6:**
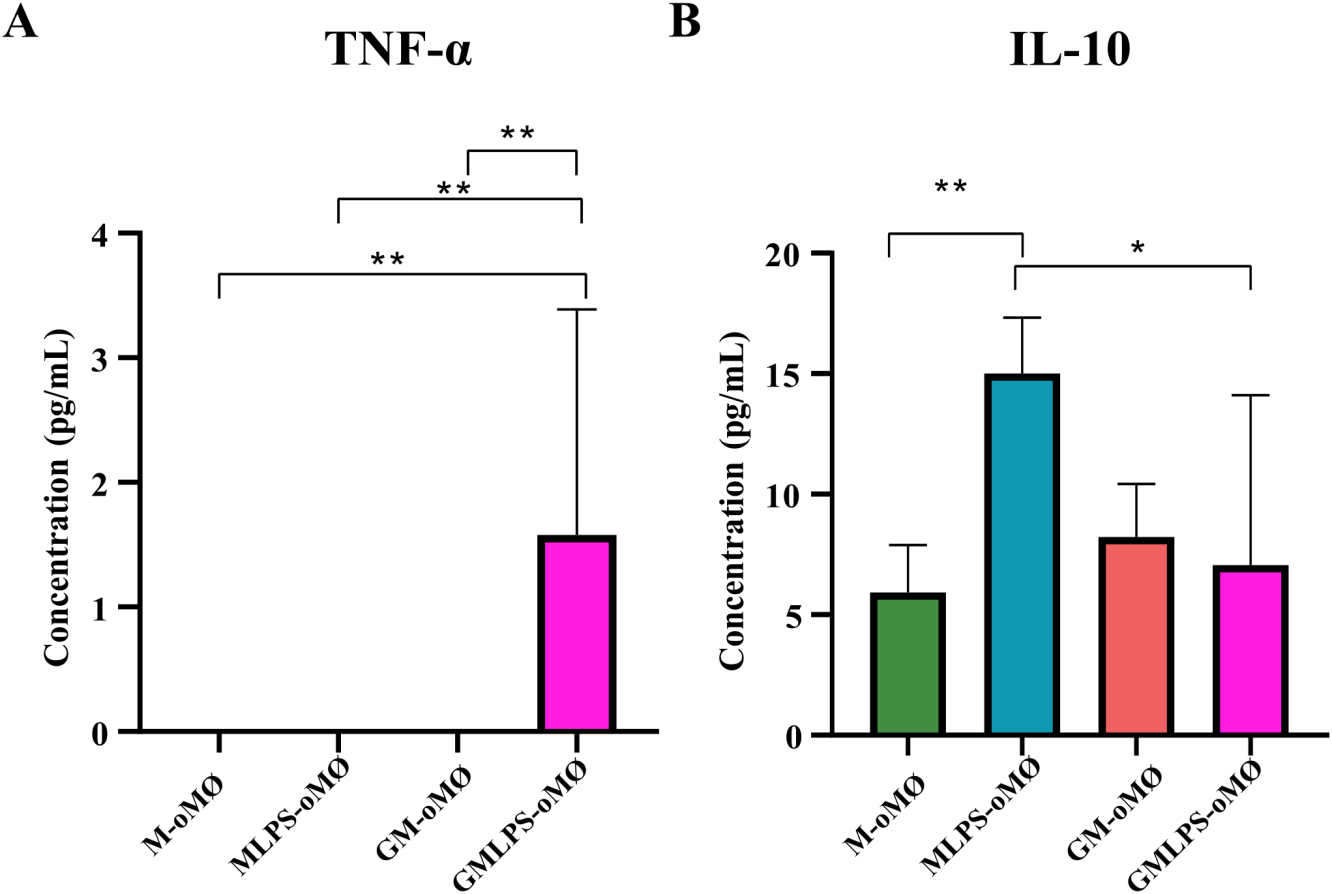
Production of TNF-α **(A)** and IL-10 **(B)** and by M- and GM-oMØs evaluated by iELISA in the absence or presence of LPS. The mean of the data is represented in a bar plot ± SD. *p<0.05. **p<0.01. N = 9 (triplicate for each condition, using cells from three different animals across independent experiments).

To functionally validate their transcriptomic differences, we assessed the production of pro- and anti-inflammatory cytokines from both macrophage subtypes upon exposure to an activating stimulus. To that end, M-oMØs and GM-oMØs were treated with 10 ng/mL LPS or left unstimulated for 16 hours. LPS stimulation promoted the release of significantly higher levels of TNF-α from GM-oMØs compared with M-oMØs (**Figure 6A**). Conversely, higher IL-10 secretion was observed in the supernatants of LPS-stimulated M-oMØs (**Figure 6B**). Consequently, LPS-activated M-oMØs consistently display a more anti-inflammatory cytokine profile than activated GM-oMØs. These findings demonstrate that LPS differentially modulates TNF-α and IL-10 secretion in GM-oMØs and M-oMØs, confirming their distinct pro-inflammatory and anti-inflammatory/regulatory transcriptional signatures.

### 3.5 Plasticity of M-oMØs in response to LPS stimulation

**Figure 7:**
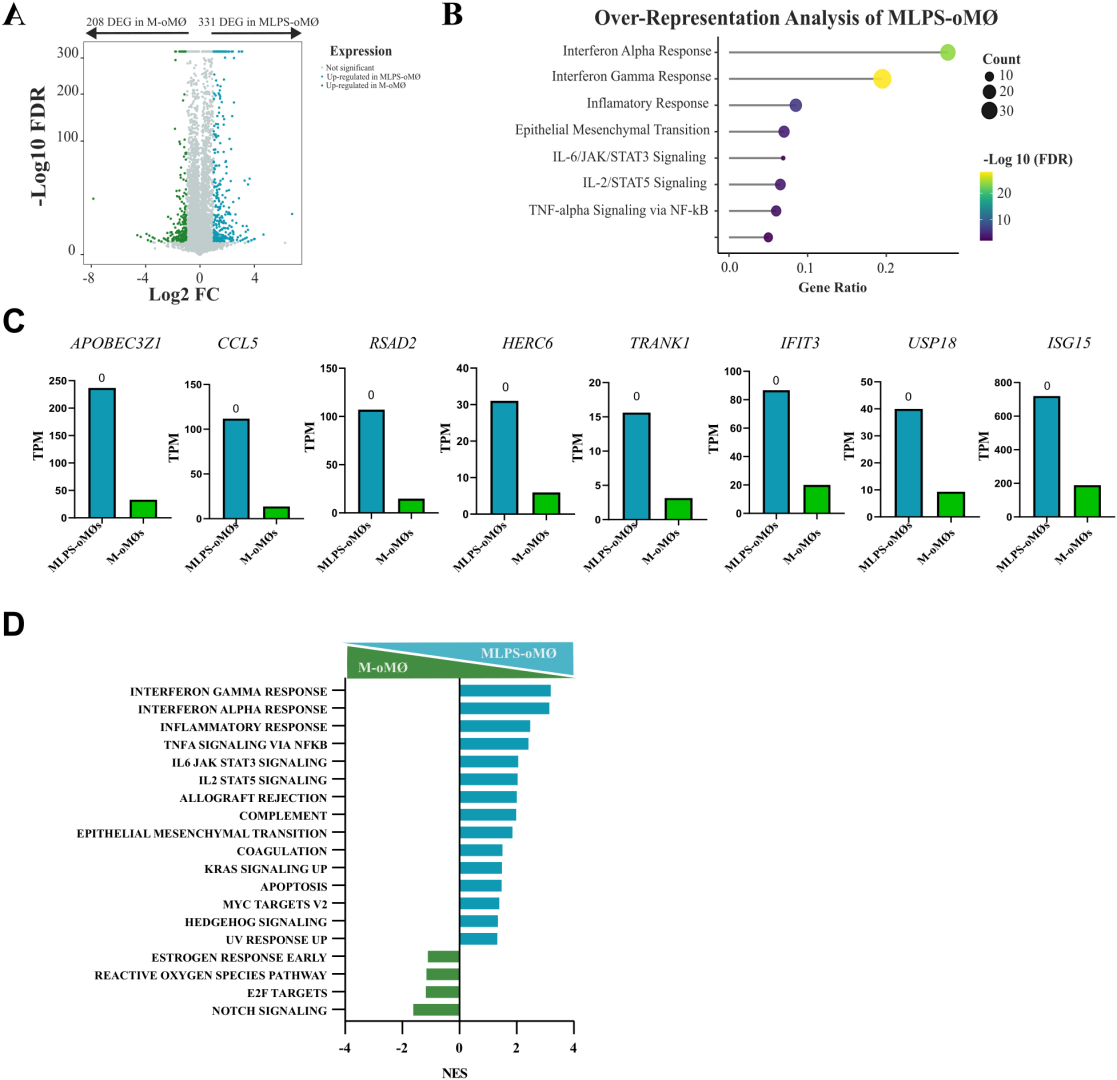
Transcriptional analysis of LPS-activated and unstimulated oMØs. **(A)** Volcano plot represents DEGs in LPS-stimulated M-oMØs (MLPS-oMØs, blue) and unstimulated M-oMØs (green) (|Log2 FC| ≥ 1, Q value ≤ 0.05) with a square transformation applied to the y-axis. **(B)** Enrichment analysis of DEGs in MLPS-oMØs. Dot size indicates the number of DEGs in each term, and the color gradient represents the -Log 10 (FDR). **(C)** Relative mRNA levels (TPM) of selected DEGs in MLPS-oMØs as determined by RNA-Seq on three independent samples. Q-value is shown in each case. **(D)** GSEA of Hallmark gene sets based on the transcriptomes of MLPS-oMØs and M-oMØs. NES are indicated for each gene set.

It has been reported that human M-CSF-dependent MØs are more plastic and represent a less differentiated state, being closer to undifferentiated monocytes than to GM-CSF-dependent MØs (4). Indeed, Van den Bossche et al. (28) demonstrated that M2-polarised monocytes retain the ability to respond to pro-inflammatory stimuli such as LPS. To corroborate whether the plasticity described in human M-MØs also exists in M-oMØs, we compared the transcriptomic profile of M-oMØs stimulated with LPS (MLPS-oMØs) for 4 hours with that of unstimulated M-oMØs. This analysis identified 539 DEGs (Q-value ≤ 0.05 and ∣Log2 FC∣≥1) between MLPS-oMØs and M-oMØs (**Supplementary Table 4**). Of these, 331 genes were significantly upregulated in MLPS-oMØs (**Figure 7A**), whose analysis revealed a significant enrichment in pathways related to Interferon Gamma Response, Interferon Alpha Response, Inflammatory Response, Epithelial Mesenchymal Transition, IL-2/STAT5 Signaling, Tumor Necrosis Factor Alpha Signaling via NF-Ƙβ, Allograft Rejection, and IL-6/JAK/STAT3 Signaling (adjusted p-value < 0.05) (**Figure 7B**). Key genes associated with Interferon Pathways included *APOBEC3Z1*, *CCL5*, *RSAD2*, *HERC6*, *TRANK1*, *IFIT3*, *USP18*, and *ISG15* (**Figure 7C**). Similarly, GSEA analysis indicated that pro-inflammatory pathways associated with Interferon Gamma Response, Interferon Alpha Response, Inflammatory Response, Tumor Necrosis Factor Alpha Signaling Via NF-Ƙβ, IL-6/JAK/STAT3 Signaling, and IL-2/STAT5 Signaling were the most significantly enriched in MLPS-oMØs (FDR < 0.05) (**Figure 7D**). These findings clearly demonstrate the significant transcriptomic plasticity of ovine M-oMØs, as LPS stimulation induced a strong shift towards a pro-inflammatory profile characterized by the robust activation of interferon and inflammatory response pathways, mirroring the previously reported responsiveness of human M-MØs to pro-inflammatory stimuli.

Finally, to complete the activation analysis, GM-oMØs were subjected to LPS stimulation. Unlike the MLPS-oMØs, the GMLPS-oMØs subset demonstrated phenotypic stability, and even increased its pro-inflammatory characteristics post-activation (**Supplementary Figure 1**). Collectively, these transcriptomic data confirm that, unlike MLPS-oMØs, the GMLPS-oMØs subset possesses a terminally defined pro-inflammatory phenotype, with LPS activation primarily serving to amplify its existing inflammatory transcriptional program rather than inducing a shift in cell fate.

## 4 Discussion

Macrophages are fundamental to both innate and adaptive immunity, with their remarkable plasticity extensively demonstrated in numerous human studies (1, 4, 9). Recent advances, such as the MoMac-VERSE classification (2), have elucidated 17 distinct human mononuclear phagocyte phenotypes with defined, conserved gene signatures, including eight specific MØ clusters associated with diverse functions in healthy and pathological states. By contrast, MØ classification in veterinary research has often been limited to an oversimplified M1/M2 dichotomy (11–13, 29, 30). However, much like in humans, this binary model is likely inaccurate given that MØ display far greater diversity and plasticity in their activation states than it can capture. Considering their importance in a wide range of small ruminant diseases, the use of primary cultures of ovine macrophages (oMØs) represents an essential tool for investigating pathogen-host interactions (11, 12, 14, 15, 31). Addressing the pressing need for a more nuanced characterization of MØs phenotypes in veterinary medicine, the present study aimed to perform, for the first time, a comprehensive immunophenotypic and functional characterization of GM-CSF- and M-CSF-differentiated oMØ populations, while also delving into the molecular underpinnings that determine these observed differences. Crucially, we further demonstrated that M-oMØs exhibit greater plasticity, rendering them more suitable for conducting pathogen-host interaction studies as they more accurately reflect the dynamic immune response induced by a pathogen in the host. This contrasts sharply with GM-CSF-differentiated macrophages, which, despite having a defined pro-inflammatory phenotype, have been the most widely used model in veterinary medicine to date (11, 12, 14).

Our study, consistent with prior research in cattle, identified three distinct monocyte subsets based on CD14 and CD16 expression: classical monocytes (CD14++CD16-), which constituted the majority (72.03%); intermediate monocytes (CD14++CD16+) (16.13%); and non-classical monocytes (CD14-CD16++) (13.06%). While these proportions broadly align with existing literature, our intermediate and non-classical monocyte populations were marginally higher than previous reports in cattle (13-17% *versus* 5-10%) (32, 33). Consistent with prior findings in cattle, our analysis in sheep identified a CD14-negative population exhibiting variable CD16 expression. Corripio Miyar et al. (33) previously characterized the analogous bovine population as NK cells (NKp46+ and CD172a−). Given that monocyte populations in ruminants have been previously characterized by others (32, 33), our current work focused solely on an initial characterization of monocyte subsets. This characterization validated that the monocyte populations utilized for subsequent macrophage differentiation were consistent with those previously described in ruminants.

After seven days in culture, M-oMØs were notably larger and produced a higher cell yield compared to the smaller, more morphologically varied GM-oMØs. This difference in yield is particularly interesting as it contrasts with reports of higher GM cell yields in human studies, potentially indicating species-specific variations or differences in culture conditions (4). Our investigation also confirmed that CD163 expression was significantly higher in M-oMØs, mirroring its established role as a key M-MØ marker in humans (34–36). Conversely, CLEC5A, a marker of human GM-MØs (34, 37, 38), proved uninformative for distinguishing ovine macrophage types, as both M-oMØs and GM-oMØs exhibited high expression. This finding diverges from human studies where CLEC5A is linked to pro-inflammatory activation (34, 35), suggesting species-specific variability in protein glycosylation that can affect marker reliability and underscoring the need to validate each marker in the target species. Therefore, further studies using a monoclonal CLEC5A antibody are warranted to fully assess its utility as a differential marker in sheep.

Our evaluation revealed that GM-oMØs exhibit a pronounced pro-inflammatory profile, marked by high RNA and protein expression of key cytokines like *TNF-α*, *IL-6*, *IL-23*, *IL-1β*, and chemokines *CCL17* and *CCL22*. Transcriptomic analysis identified 408 upregulated genes in GM-oMØs, predominantly associated with interferon pathways (e.g., *IFI30*, *TRIM14*, *IFI3*, *IFI2*, *IRF7*), a finding rigorously confirmed by enrichment and GSEA analyses. These results consistently align with the established pro-inflammatory characteristics of human GM-MØs (4, 6, 7, 34–36, 39). Comparative analysis with the human MoMac-VERSE (2) demonstrated that GM-oMØs largely correspond to Cluster#4 (ISG_Mo), an interferon-stimulated gene-rich monocyte population with an M1-like inflammatory signature, implying crucial roles in antiviral defense and pro-inflammatory responses (2, 7, 40). In addition, LPS stimulation resulted in elevated pro-inflammatory activity (**Supplementary Figure 1**). The subsequent transcriptomic analysis of GMLPS-oMØs identified 148 upregulated genes predominantly linked to inflammatory pathways (e.g. *CSF2, CSF3, IL23A, CCL20, PTX3*), mirroring findings in human macrophages. This phenotypic persistence strongly supports the view (4, 28) that pro-inflammatory macrophage subsets exhibit limited phenotypic plasticity, consistent with a terminal differentiation state. This alignment is reinforced by the greater epigenetic drift observed in GM-CSF-polarized monocytes (4). On the other hand, M-oMØs consistently exhibited a regulatory and anti-inflammatory profile, predominantly characterized by high *IL-10* expression. Our transcriptomic analysis identified 248 upregulated genes in M-oMØs, many of which (e.g., *RGS20*, *CLEC1A*, *TBC1D16*, *ARL8A*, *BCL6*) are known to be involved in cellular homeostasis and immune regulation. Notably, several of these genes are recognized as human M-macrophage markers (7, 36, 39, 41–47). Furthermore, GSEA analysis revealed enrichment in pathways such as Notch, Apical Junctions, and WNT Beta Catenin signaling. Comparative analysis with the human MoMac-VERSE atlas (2) showed that M-oMØs significantly aligned with anti-inflammatory clusters (Cluster #2, #3, and #16), thereby reinforcing their anti-inflammatory nature. However, certain associations with human tumor-associated macrophage clusters (Cluster #7, #11 and #14) might reflect genuine species divergence or contextual differences. Crucially, LPS activation dramatically shifted the M-oMØ phenotype towards a pro-inflammatory state, unequivocally demonstrating their substantial plasticity. This responsiveness, which mirrors that of human M-CSF-polarized monocytes, suggests that M-oMØs exist in a less terminally differentiated state. This heightened plasticity in humans has been attributed to less extensive epigenetic rewiring compared to GM-MØs, or even to metabolic differences such as mitochondrial dysfunction observed in pro-inflammatory states (4, 28).

**Figure 8:**
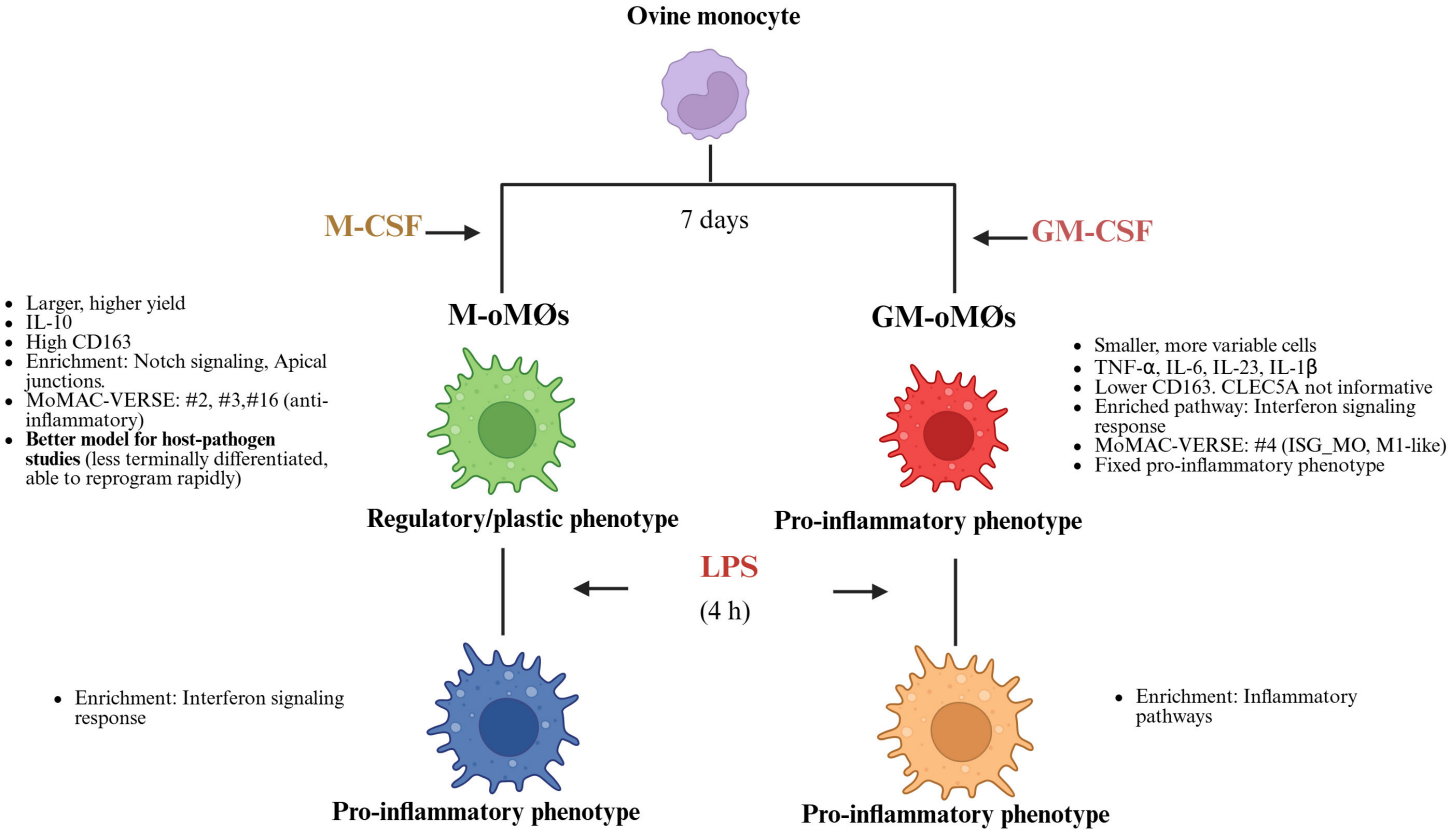
Schematic representation illustrating the key differences in phenotype, function, and plasticity between M-oMØs and GM-oMØs. Created in BioRender. Amieva, R (2025). https://BioRender.com/p98uwic.

The authors acknowledge two primary constraints of the present study. First, the use of only three donor animals (N = 3) warrants careful consideration, as this restricted sample size limits the statistical power, reproducibility, and generalizability of the results. Second, the observed low and variable detection levels for IL-10 and TNF-alpha in LPS-stimulated M and GM- oMØs suggest a necessary future optimization. Since oMØs may differ from human MØs, these findings indicate that an LPS concentration greater than 10 ng/mL is probably required to induce robust protein expression. In summary, our study provides the first comprehensive immunophenotypic, transcriptomic, and functional characterization of ovine monocyte-derived macrophages. We rigorously demonstrate that GM-oMØs inherently possess a defined pro-inflammatory phenotype, while non-activated M-oMØs display a distinct regulatory and anti-inflammatory profile (**Figure 8**). Importantly, our findings reveal the remarkable transcriptomic plasticity of M-oMØs, indicating their swift acquisition of a pro-inflammatory signature upon LPS stimulation, consistent with human macrophage behavior. This work fundamentally challenges the oversimplified M1/M2 classification in ovine immunology and underscores the vital importance of selecting appropriate macrophage models for *in vitro* studies. By offering a more nuanced understanding of oMØ subsets and their dynamic responses, our research provides a robust foundation for future investigations on ovine host defense mechanisms, vaccine development, and disease pathogenesis, paving the way for more accurate and translatable findings in veterinary immunology.

## Data Availability

The datasets presented in this study can be found in online repositories. The names of the repository/repositories and accession number(s) can be found in the article/**Supplementary Material**. RNA-seq data were deposited in the European Nucleotide Archive (ENA) (PRJEB93905).

## Supplementary material

Supplementary Figure 1 Transcriptional analysis of GMLPS-activated and unstimulated GM-oMØs. **(A)**. Volcano plot represents DEGs in LPS-stimulated GM-oMØs (GMLPS-oMØs, orange) and unstimulated M-oMØs (red) (|Log2 FC| ≥ 1, Q value ≤ 0.05) with a square transformation applied to the y-axis. **(B).** Enrichment analysis of DEGs in GMLPS-oMØs. Dot size indicates the number of DEGs in each term, and the color gradient represents the -Log 10 (FDR). **(C).** Relative mRNA levels (TPM) of selected DEGs in GMLPS-oMØs as determined by RNA-Seq on three independent samples. Q-value is shown in each case.
