# Supplementary material for: Ovine macrophage identity and plasticity: novel insights into CSF-driven polarization and species-specific responses
Source: Front Immunol. 2025 Nov 25;16:1680086. doi: 10.3389/fimmu.2025.1680086 (PMC12685658; doi:10.3389/fimmu.2025.1680086)
Supplement: Supplementary file 1 [file Table1.docx]

**Supplementary Table 1:** List of antibodies used for immunophenotypic analysis of ovine monocyte and macrophages.

| **Antibody** | **Conjugate** | **Supplier** | **Species** | **Type** | **Clone** | **Reference** |
| --- | --- | --- | --- | --- | --- | --- |
| **CD14** | FITC | Bio-Rad Laboratories (Pleasanton, USA) | Mouse anti-human | Monoclonal | TÜK4 | MCA1568F |
| **CD16** | Alexa 647 |  |  |  | KD1 | MCA5665A647 |
| **MHC Class II** | RPE |  | Mouse anti-ovine |  | 49.1 | MCA2228F |
| **CD80** | RPE |  | Mouse anti-bovine |  | IL-A159 | MCA2436PE |
| **CD86** | RPE |  |  |  | IL-A190 | MCA2437PE |
| **CD172a** | RPE-Cy5 |  |  |  | CC149 | MCA2041C |
| **CD11b** | FITC |  |  |  | CC126 | MCA1425F |
| **CD163** | Startbright blue 700 |  | Mouse anti-human |  | EDHu-1 | MCA1853SBB700 |
| **CLEC5A** | Alexa 647 | Bioss Antibodies (USA) |  | Polyclonal | - | bs-2663R |
| **Viability staining solution** | 7-AAD | Biolegend | - | - | - | 420404 |
