# Supplementary material for: Ovine macrophage identity and plasticity: novel insights into CSF-driven polarization and species-specific responses
Source: Front Immunol. 2025 Nov 25;16:1680086. doi: 10.3389/fimmu.2025.1680086 (PMC12685658; doi:10.3389/fimmu.2025.1680086)
Supplement: Supplementary file 2 [file Table2.docx]

**Supplementary Table 2**: Sequences of primers used for cytokine real-time PCR (qPCR) and standard curve data.

| **Target** | **Primer** | **Primer sequence (5’-3’)** | **Product size (bp)** | **References** |
| --- | --- | --- | --- | --- |
| IL-10 | IL10-Up | TGCTGGATGACTTTAAGGGTTACC | 60 | (1) |
|  | IL10-Rp | AAAACTGGATCATTTCCGACAAG |  |  |
| TNF-α | TNF-Up | CCAGAGGGAAGAGCAGTCC | 86 |  |
|  | TNF-Rp | GGAGCGCTGATGTTGGCTAC |  |  |
| IL-6 | IL6-Up | CTGGGTTCAATCAGGCGATT | 150 |  |
|  | IL6-Rp | GGATCTGGATCAGTGTTCTGA |  |  |
| TGF-β | TGF-Up | GGTGGAATACGGCAACAAAA | 117 |  |
|  | TGF-Rp | CGAGAGAGCAACACAGGTTC |  |  |
| β-actin | βactin-Up | ACACCGCAACCAGTTCGCCAT | 216 |  |
|  | βactin-Rp | GTCAGGATGCCTCTCTTGCT |  |  |
| IL-23 | IL23-Up | AGGGAACTCTTGCAGCCCAA | 227 | (2) |
|  | IL23-Rp | TAGCATTACTGAGCCACGGAGG |  |  |
| IL-1β | IL1-Up | ATGGGTGTTCTGCATGAG | 60 | (3) |
|  | IL1-Rp | AAGGCCACAGGAATCTTG |  |  |
| IL-12 | IL12-Up | AGTACACAGTGGAGTGTCAG | 157 | (4) |
|  | IL12-Rp | TTCTTGGGTGGGTCTGGTTT |  |  |
| CXCL10 | CXCL10-Up | CACTCCTCAACTCTTCAGGC | 262 | (5) |
|  | CXCL10-Rp | CCATTCCTTTTCATTGTGGC |  |  |
| CCL22 | CCL22-Up | CGGGACTACATCCGTTACCC | 121 | (6) |
|  | CCL22-Rp | CAGCACAGATCTCTCGGTCC |  |  |
| CCL24 | CCL24-Up | GCAGGAGTGATCTTCACCACC | 115 |  |
|  | CCL24-Rp | TAGCGGAGGCTTTCTTCTGC |  |  |
| GAPDH | GAPDH-Up | GGTGATGCTGGTGCTGAGTA | 56 |  |
|  | GAPDH-Rp | TCATAAGTCCCTCCACGATG |  |  |
| CCL17 | CCL17-Up | CCATTCCCAAAAAGGCGCTC | 147 | This work |
|  | CCL17-Rp | TCTGCAAGTACCTCACTGCC |  |  |

1. Arranz-Solís D, Benavides J, Regidor-Cerrillo J, Horcajo P, Castaño P, del Carmen Ferreras M, Jiménez-Pelayo L, Collantes-Fernández E, Ferre I, Hemphill A, et al. Systemic and local immune responses in sheep after *Neospora caninum* experimental infection at early, mid and late gestation. *Vet Res* (2016) 47:2. doi: 10.1186/s13567-015-0290-0

2. Sassu EL, Kangethe RT, Settypalli TBK, Chibssa TR, Cattoli G, Wijewardana V. Development and evaluation of a real-time PCR panel for the detection of 20 immune markers in cattle and sheep. *Vet Immunol Immunopathol* (2020) 227:110092. doi: 10.1016/j.vetimm.2020.110092

3. Fach SJ, Meyerholz DK, Gallup JM, Ackermann MR, Lehmkuhl HD, Sacco RE. Neonatal ovine pulmonary dendritic cells support bovine respiratory syncytial virus replication with enhanced interleukin (IL)-4 And IL-10 gene transcripts. *Viral Immunol* (2007) 20:119–130. doi: 10.1089/vim.2006.0056

4. Rico-San Román L, Amieva R, Horcajo P, García-Sánchez M, Pastor-Fernández I, Ortega-Mora LM, Collantes-Fernández E. Characterization of *Neospora caninum* virulence factors NcGRA7 and NcROP40 in bovine target cells. *Vet Parasitol* (2023) 320:109973. doi: 10.1016/j.vetpar.2023.109973

5. Imakawa K, Imai M, Sakai A, Suzuki M, Nagaoka K, Sakai S, Lee S-R, Chang K-T, Echternkamp SE, Christenson RK. Regulation of conceptus adhesion by endometrial CXC chemokines during the implantation period in sheep. *Mol Reprod Dev* (2006) 73:850–858. doi: 10.1002/mrd.20496

6. Gossner A, Hassan MA. Transcriptional analyses identify genes that modulate bovine macrophage response to *Toxoplasma* infection and immune stimulation. *Front Cell Infect Microbiol* (2020) 10:437. doi: 10.3389/fcimb.2020.00437
