# Supplementary material for: Ovine macrophage identity and plasticity: novel insights into CSF-driven polarization and species-specific responses
Source: Front Immunol. 2025 Nov 25;16:1680086. doi: 10.3389/fimmu.2025.1680086 (PMC12685658; doi:10.3389/fimmu.2025.1680086)
Supplement: Supplementary file 3 [file Table3.docx]

**Supplementary Table 3:** Immunophenotypic analysis by flow cytometry of M-oMØs and GM-oMØs. This table presents the individual replicate values for both the percentage of positive cells and the Mean Fluorescence Intensity (MFI).

|  |  | **CD14+**  **(%)** | **MFI**  **CD14+** | **CD16+**  **(%)** | **MFI CD16+** | **CD11b+**  **(%)** | **MFI CD11b+** | **CD80+**  **(%)** | **MFI CD80+** | **CMH II+**  **(%)** | **MFI CMHII+** | **CD86+**  **(%)** | **MFI CD86+** | **CD163+ (%)** | **MFI CD163+** | **CLEC5A+ (%)** | **MFI CLEC5A+** | **CD172a+**  **(%)** | **MFI CD172+** |
| --- | --- | --- | --- | --- | --- | --- | --- | --- | --- | --- | --- | --- | --- | --- | --- | --- | --- | --- | --- |
| M-oMØs | R1 | 96.5 | 130 | 95.7 | 36.2 | 94.5 | 259 | 83.8 | 69.8 | 36.3 | 30.5 | 15.7 | 30.5 | 48 | 24 | 95.7 | 28.9 | 86.3 | 61.5 |
|  | R2 | 98.6 | 44.1 | 95.6 | 22.9 | 95.7 | 37.9 | 32.1 | 20 | 18.2 | 13 | 8.49 | 12.5 | 40.5 | 17.9 | 97.2 | 41 | 45.8 | 24.9 |
|  | R3 | 89.7 | 40 | 90.6 | 33.7 | 84.7 | 48.7 | 39.3 | 22.7 | 18.3 | 21.3 | 6.1 | 17.5 | 56.4 | 31 | 86.9 | 19.3 | 32.8 | 33 |
| GM-oMØs | R1 | 95.2 | 235 | 94.4 | 42.6 | 91.6 | 346 | 85.9 | 90.6 | 18.3 | 26.9 | 12.4 | 30 | 28 | 20 | 92.2 | 20 | 57.6 | 30.2 |
|  | R2 | 98.8 | 81.3 | 93.6 | 29.2 | 94 | 64.9 | 55.4 | 19.8 | 19.1 | 12.7 | 4 | 12.3 | 22.4 | 16 | 95.7 | 26.9 | 51 | 19.3 |
|  | R3 | 92.4 | 66.7 | 91.6 | 43.7 | 88.8 | 89.8 | 74.9 | 27.9 | 18 | 28.6 | 9.15 | 17.6 | 35.5 | 24.1 | 87.5 | 24.4 | 40.1 | 28.2 |

R: replicate; MFI: mean fluorescence intensity
