# Supplementary figures and images for: Ovine macrophage identity and plasticity: novel insights into CSF-driven polarization and species-specific responses
Source: Front Immunol. 2025 Nov 25;16:1680086. doi: 10.3389/fimmu.2025.1680086 (PMC12685658; doi:10.3389/fimmu.2025.1680086)

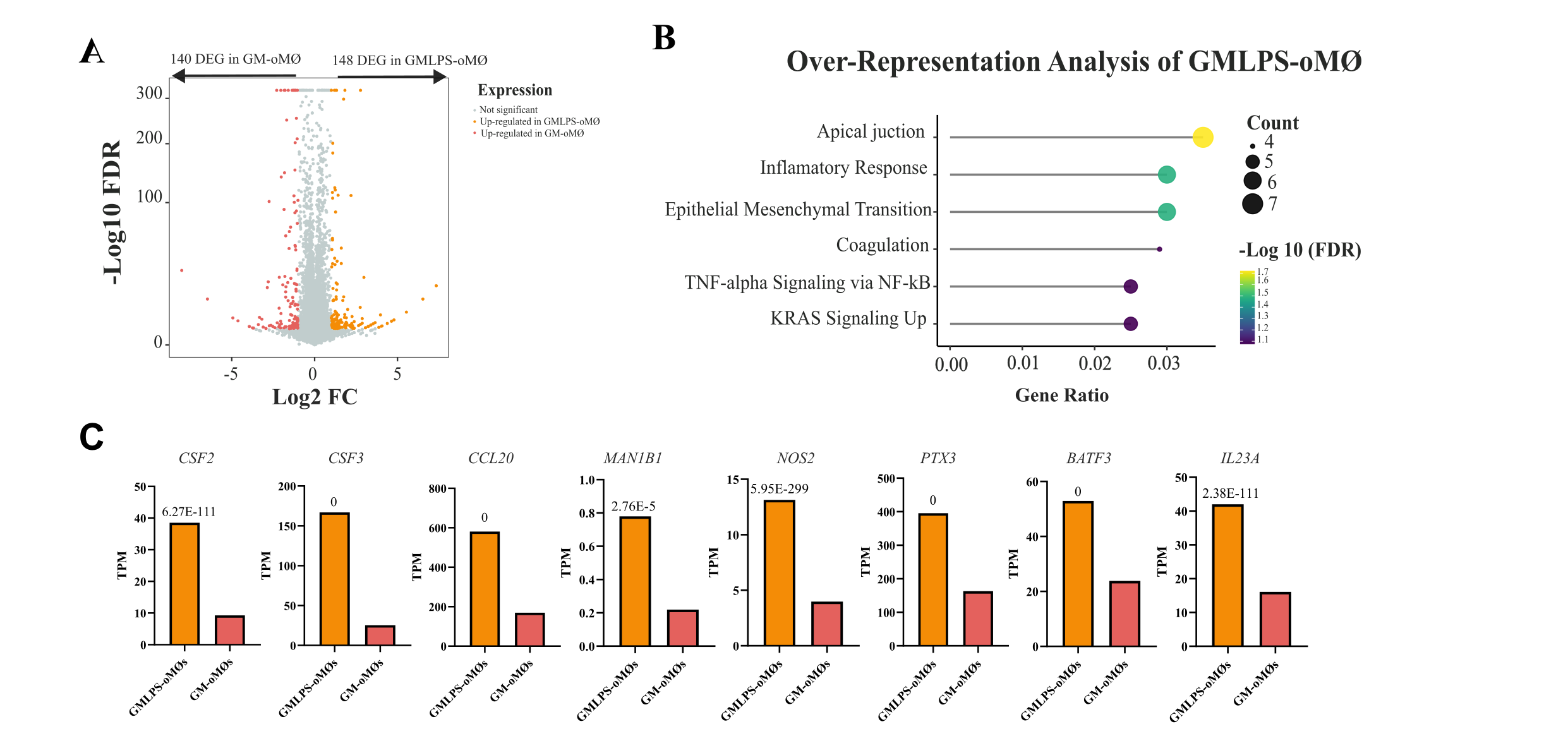

Supplement: Supplementary Figure 1 — Transcriptional analysis of GMLPS-activated and unstimulated GM-oMØs. (A). Volcano plot represents DEGs in LPS-stimulated GM-oMØs (GMLPS-oMØs, orange) and unstimulated M-oMØs (red) (|Log2 FC| ≥ 1, Q value ≤ 0.05) with a square transformation applied to the y-axis. (B). Enrichment analysis of DEGs in GMLPS-oMØs. Dot size indicates the number of DEGs in each term, and the color gradient represents the -Log 10 (FDR). (C). Relative mRNA levels (TPM) of selected DEGs in GMLPS-oMØs as determined by RNA-Seq on three independent samples. Q-value is shown in each case. [file Image1.tiff]
